# Bacterial isolates and their antimicrobial susceptibility profile of superficial and deep-seated skin and soft tissue infections

**DOI:** 10.2478/abm-2023-0045

**Published:** 2023-09-17

**Authors:** Rao Muhammad Abid Khan, Sunil Kumar Dodani, Ali Nadeem, Sana Jamil, Mirza Naqi Zafar

**Affiliations:** Department of Microbiology, Sindh Institute of Urology and Transplantation, Karachi 74200, Pakistan; Department of Infectious Diseases, Sindh Institute of Urology and Transplantation, Karachi 74200, Pakistan; Department of Biochemistry, Sindh Institute of Urology and Transplantation, Karachi 74200, Pakistan

**Keywords:** bacteria, drug resistance, oral, prevalence, skin, soft tissue

## Abstract

**Background:**

Skin and soft tissue infections (SSTIs) are caused by microbial invasion of healthy or damaged skin. SSTIs are difficult to manage and contribute to chronicity and emergence of antimicrobial resistance.

**Objectives:**

To ascertain the prevalence of bacteria causing SSTIs and their antimicrobial susceptibility patterns.

**Methods:**

A prospective study between November 2020 and May 2021. A total of 447 samples from SSTIs were analyzed.

**Results:**

A total of 347 samples revealed mono-bacterial growth, of which 67% were male. SSTIs are common among patients aged 21–50 years with the dominance (78%) of gram-negative rods (GNRs). *Escherichia coli* (36%), *Klebsiella* spp. (22%), *Staphylococcus aureus* (16%), and *Pseudomonas aeruginosa* (11%) were predominant organisms. GNRs were highly resistant (>65%) to ciprofloxacin and trimethoprim–sulfamethoxazole. For injectable antibiotics, the highest resistance was determined against ceftriaxone, and the least resistance was determined against amikacin. Resistance against carbapenem was the highest among *P. aeruginosa* (53%) and *Klebsiella* spp. (32%). *S. aureus* showed the highest resistance against ciprofloxacin, and the least resistance was determined against clindamycin. Of 57 *S. aureus* isolates, 86% isolates were methicillin-resistant *Staphylococcus aureus* (MRSA). All isolates of *P. aeruginosa* and *S. aureus* were sensitive to polymyxin B and vancomycin, respectively. The prevalence of multidrug-resistant *E. coli* and *Klebsiella* spp. was higher among deep-seated SSTIs (dSSTIs).

**Conclusions:**

The predominant etiology of SSTIs is GNR. Currently, there is very high resistance against oral antibiotics. Antimicrobial resistance against carbapenem has also increased. Moreover, there is a high frequency of MRSA. MDR *E. coli* and *Klebsiella* spp. isolates are frequently involved in dSSTIs.

Skin and soft tissue infections (SSTIs) have been defined as invasion of the epidermis, dermis, and subcutaneous tissue by bacteria. The term SSTI covers infections of all anatomical and surgical sites where the integrity of the skin has been breached [1]. If SSTIs are just presented with skin redness, they are called mild or superficial SSTIs (sSSTIs), and if present along with systemic signs such as tachycardia, fever, and hypotension, they are classified as deep-seated SSTIs (dSSTIs) [2]. The Infectious Diseases Society of America (IDSA) classifies SSTIs into 2 categories: purulent infections (e.g., furuncles, carbuncles, and abscesses) and non-purulent infections (e.g., erysipelas, cellulitis, and necrotizing fasciitis) [1]. SSTIs are usually associated with prolonged hospital stay, increased medical cost, and diverse etiology. The management of dSSTIs is challenging and often requires hospitalization, incision, drainage, or debridement [3]. Predisposing factors include old age, trauma, cardio-pulmonary or hepato-renal disease, diabetes mellitus, and immunosuppression [4].

Although SSTIs affect all age groups, they are more frequent among men aged <5 years and >65 years, involving the lower leg region [5]. Annual outpatient visits for SSTIs are estimated at >14 million/year in the United States [2]. Nearly 7%–10% of admitted patients are affected by SSTIs, and they represent the third most common diagnosis after chest pain and asthma in emergencies [6].

SSTIs can be mono-microbial or poly-microbial. Methicillin-resistant *Staphylococcus aureus* (MRSA) has been accounted for 59% of SSTIs. Although gram-negative rods (GNRs) are often overlooked as a cause of SSTIs, several studies claim GNRs to be the dominant cause [7,8,9]. Poly-microbial infections account for 20%–30% [10].

Similarities in clinical presentation and low probability of isolating an organism make the diagnosis challenging [5]. Treatment decisions are often empirical and based on educational guess, host characteristics, likely pathogens, and local susceptibility patterns [5]. Treatments are frequently topical, oral, intravenous, or surgeries [11]. Oral formulations are preferred to avoid higher cost and adverse effects of intravenous administration. Intravenous treatment is reserved for inpatients, for life-threatening dSSTIs, or for those who are unable to absorb oral formulations or fail to achieve adequate concentration at the infection site [12].

The increase in antibiotic resistance and its rapid spread among pathogens has posed a threat to public health worldwide [10]. The growing prevalence of multidrug-resistant pathogens like MRSA and carbapenem-resistant enterobacteriaceae (CRE) is a global concern [9]. Although few studies from Pakistan report the incidence rate and antimicrobial susceptibility profile of *Staphylococcus aureus (S. aureus)* causing SSTIs, the data for other microbes are limited [11, 13,14,15,16,17].

## Methods

Ethical approval was obtained from the Ethical Review Committee of the Sindh Institute of Urology and Transplantation (approval No. SIUT-ERC-2020/A-244). Because this was a laboratory-based study, the ERC had exempted this research from obtaining informed consent from patients.

### Study design

A prospective laboratory-based study in the Department of Microbiology, Sindh Institute of Urology and Transplantation (SIUT), Pakistan, conducted from November 2020 to May 2021.

### Study population

A total of 447 patients of all ages and both sexes attending out-patient clinics or admitted in wards with SSTIs were included. A physician recorded demographic characteristics and underlying cause and stratified the SSTIs into sSSTIs and dSSTIs.

### Inclusion criteria

Mono-bacterial growth either gram-positive cocci (GPC) or GNRs. We used the STROBE cross-sectional checklist to report our results.

### Exclusion criteria

Transplant and burns patients, poly-microbial growth, yeasts/fungus, gram-positive rods, and gram-negative cocci.

### Sample collection and processing

Pus swabs, discharge/secretion, exudates, aspirate, or tissue samples from SSTIs were inoculated on the chocolate, blood, and MacConkey agar (Oxoid Ltd, Hampshire, UK).

### Identification and antibiotic susceptibility testing

Bacteria were identified using standard methods, such as Gram staining, catalase, coagulase, oxidase, mannitol salt agar (MSA), DNAse, bile esculin agar, and fermentation of different sugars. Antimicrobial susceptibility was determined for only top 4 most prevalent organisms using Kirby–Bauer disk diffusion method against the following antibiotics:

(ORAL) trimethoprim–sulfamethoxazole (SXT) (25 μg), ciprofloxacin (5 μg), amoxicillin + clavulanic acid (AMC) (30 μg), erythromycin (15 μg), clindamycin (2 μg), (injectable) ceftriaxone (30 μg), amikacin (30 μg), tazobactam + piperacillin (TZP) (110 μg), sulbactam + cefoperazone (SCF) (105 μg), and (carbapenem) imipenem (10 μg).

Susceptibility to polymyxin B and vancomycin was determined using the microbroth dilution method and E test, respectively. Polymyxin B MIC interpretation was done as intermediate = 2 μg/mL and resistant = 4 μg/mL. A cefoxitin 30 μg disk was used to determine MRSA. The SCF zone diameter of ≤15 mm was interpreted as resistant, 16–20 mm as intermediate, and ≥21 mm as sensitive [18]. The Clinical Laboratory Standard Institute (CLSI) M100 guidelines were used to interpret the results [19].

### Multidrug resistance

Multidrug resistance (MDR) is defined as bacterial resistance to at least 1 agent in 3 or more antimicrobial classes [7].

### Data analysis

Data analysis was performed using MS excel, SPSS version 20, and GraphPad prism 9.3.1.

## Results

A total of 447 specimens from 447 SSTI patients ranging in age from 1 to 78 years, with mean age of 33.76 ± 21.27 years, were included in this study. Of these, mono-microbial growth was isolated from 347 (78%) patients, of which 61% were inpatients and 39% were outpatients.

Of 347 patients, 67% were male and 33% were female. Most of the patients (157 (45%)) were in the 21–50 years age group, 26% were older than 51 years, and 18% were in the 1–20 years age group. The age data were missing for 38 (11%) patients **(Table 1)**.

**Table 1.** Demographic characteristics and distribution of cases among inpatients and outpatients suffering from sSSTIs and dSSTIs

	**sSSTI n = 124 (36%)**		**dSSTI n = 223 (64%)**		**Total n = 347**	

	**Frequency**	**%**	**Frequency**	**%**	**Frequency**	**%**
**Patient setting**						
Inpatient	63	30	148	70	211	61
Outpatient	61	45	75	55	136	39
**Gender**						
Male	88	38	144	62	232	67
Female	36	31	79	69	115	33
**Age (years)**						
≤20	23	38	38	62	61	18
21–50	56	36	101	64	157	45
≥51	35	38	56	62	91	26
**Underlying medical condition**						
Chronic kidney disease	47	37	80	63	127	37
Diabetes mellitus	36	39	56	61	92	27
Malignancy	13	28	33	72	46	13
Immunosuppression	16	42	22	58	38	11
Chronic liver disease	9	28	23	72	32	9
Heart failure	3	25	9	75	12	3
**SSTIs**						
Wound infection	82	66	-	-	-	-
Diabetic foot ulcer	15	12	-	-	-	-
Impetigo	15	12	-	-	-	-
Cellulitis	12	10	-	-	-	-
Abscess	-	-	138	62	-	-
Surgical site infection	-	-	72	32	-	-
Necrotizing fasciitis	-	-	13	6	-	-
**Organisms**						
*E. coli*	57	45	69	55	126	36
*Klebsiella* spp.	27	35	49	65	76	22
*S. aureus*	14	24	43	76	57	16
*Pseudomonasaeruginosa*	9	24	29	76	38	11
*Proteus* spp.	7	58	5	42	12	3
*Acinetobacter* spp.	5	45	6	55	11	3
*Morganella morganii*	3	30	7	70	10	3
CoNS	-	-	6	100	6	2
*Enterococcus* spp.	2	40	3	60	5	1.4
*Streptococcus* spp.	-	-	4	100	4	01
*Aeromonas hydrophila*	-	-	1	100	1	0.2
*Streptococcuspneumoniae*	-	-	1	100	1	0.2

CoNS, coagulase-negative *Staphylococcus*; SSTIs, skin and soft tissue infections; sSSTIs, superficial skin and soft tissue infections; dSSTIs, deep-seated skin and soft tissue infections.

**Figure 1.**
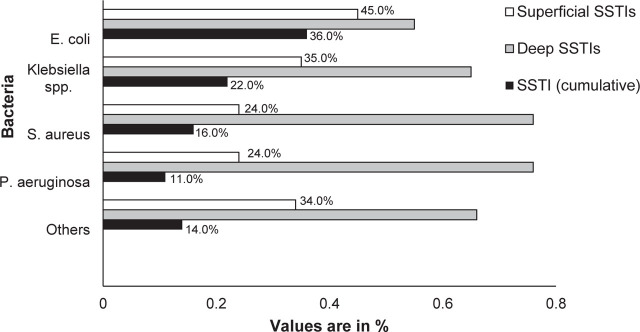
Prevalence of bacteria causing superficial and deep-seated SSTIs among patients attending the SIUT Hospital from November 2020 to May 2021. Others = *Proteus* spp., *Acinetobacter* spp., *Morganella morganii, Aeromonas hydrophila*, CoNS, *Enterococcus* spp., *Streptococcus* spp., and *Streptococcus pneumoniae*. CoNS, coagulase-negative *Staphylococcus.*

The prevalence of *E. coli, Klebsiella* spp., *Pseudomonas aeruginosa* and *S. aureus* was significantly higher among dSSTIs **(Figure 1)**.

Most of the samples in our study were dSTTIs (64%). The common sites among dSTTIs were abscess (62%) and surgical sites (32%), and the least common was necrotizing fasciitis (6%). Wound (66%) was the most common representative site for sSSTIs, followed by diabetic foot ulcer and impetigo (12% each), and cellulitis (10%). The most common underlying condition was chronic kidney disease (37%), followed by diabetes mellitus (27%), malignancy (13%), immunosuppression (11%), chronic liver disease (9%), and heart failure (3%) **(Table 1)**. The prevalence of GNRs was higher (274 (79%)) among SSTIs than GPC. The most common isolated GNRs were *E. coli* (36%), *Klebsiella* spp. (22%), and *Pseudomonas aeruginosa* (11%). The most common GPC was *S. aureus* (16%) **(Figure 1)**.

A number of other GNRs including *Proteus* spp. (3%), *Acinetobacter* spp. (3%), *M. morganii* (3%), and *Aeromonas hydrophila* (0.2%), and GPC including coagulase-negative *Staphylococcus* (CoNS) (2%), *Enterococcus* spp. (1.4%), *Streptococcus* spp. (1%), and *S. pneumoniae* (0.2%) were also isolated. We have limited our results and discussion to only top 4 prevalent GNRs and GPC isolates (297) (86%) (i.e., *E. coli, Klebsiella* spp., *P. aeruginosa*, and *S. aureus*) in this study.

For oral antibiotics, all 3 GNRs revealed a high level of resistance (>65%) against ciprofloxacin and SXT. *Klebsiella* spp. and *E. coli* showed resistance against AMC (62% and 49%), respectively.

For injectable antibiotics, the least level of resistance among each GNR was determined against amikacin, and the highest was determined against ceftriaxone. *P. aeruginosa* and *Klebsiella* spp. were highly resistant to SCF (42% and 49% respectively) and TZP (34% and 43%,). *E. coli* showed the highest resistance against SCF (30%) and TZP (29%), and the least resistance against amikacin (9%). None of the *P. aeruginosa*isolates showed resistance against polymyxin B.

For carbapenem, *P. aeruginosa* showed the highest resistance (53%), followed by *Klebsiella* spp. (32%) and *E. coli* (18%). *S. aureus* showed the highest resistance against ciprofloxacin (91%), followed by AMC (81%), erythromycin (68%), and SXT (42%) and the least resistance against clindamycin (14%). The overall prevalence of MRSA was 86%. The prevalence of MRSA among superficial and deep-seated SSTIs was 11 (78.5%) and 38 (88%), respectively. None of the *S. aureus* isolate was resistant to vancomycin. *E. coli* and *Klebsiella* spp. isolates from dSSTIs were comparatively more resistant to oral, injectable, and carbapenem antibiotics. Conversely, *S. aureus* and *P. aeruginosa* isolates from sSSTIs were comparatively more resistant to oral, injectable, and carbapenem antibiotics **(Table 2)**.

**Table 2.** Antimicrobial resistance pattern of common bacteria isolated from sSSTIs and dSSTIs against oral, injectable, and carbapenem antibiotics

**Bacterial isolate**	**SSTIs**	**Number of resistance pathogens to antimicrobial agents, n (%)**									

		**P.O.**	**Injectable**								**Carbapenem**

		**SXT**	**CIP**	**AMC**	**E**	**DA**	**AK**	**TZP**	**SCF**	**CRO**	**IPM**
*E. coli* (n = 126)	sSSTI (n = 57)	43 (75.4)	42 (73.6)	22 (38.5)	-	-	2 (3.5)	14 (24.5)	13 (22.8)	38 (66.7)	9 (15.7)
	dSSTI (n = 69)	48 (70)	56 (81)	40 (58)	-	-	9 (13)	23 (33)	25 (36)	61 (88)	14 (20)
*Klebsiella* spp. (n = 76)	sSSTI (n = 27)	16 (59)	21 (77.8)	17 (63)	-	-	9 (33.3)	9 (33.3)	10 (37)	22 (81.4)	7 (25.9)
	dSSTI (n= 49)	38 (78)	40 (82)	30 (61)	-	-	20 (41)	24 (49)	27 (55)	43 (88)	17 (35)
*P. aeruginosa* (n = 38)	sSSTI (n = 9)	9 (100)	6 (66.6)	-	-	-	4 (44.4)	4 (44.4)	5 (55.5)	-	6 (66.6)
	dSSTI (n = 29)	26 (90)	20 (69)	-	-	-	11 (38)	9 (31)	11 (38)	-	14 (48)
*S. aureus* (n = 57)	sSSTI (n = 14)	8 (57.1)	13 (92.8)	11 (78.5)	10 (71.4)	3 (21.4)	-	-	-	-	-
	dSSTI (n= 43)	16 (37)	39 (91)	35 (81)	29 (67)	5 (12)	-	-	-	-	-

−, not done; AK, amikacin; AMC, amoxicillin + clavulanic acid; CIP, ciprofloxacin; CRO, ceftriaxone; DA, clindamycin; dSSTIs, deep-seated skin and soft tissue infections; E, erythromycin; IPM, imipenem; n, number of isolates; P.O., Per os; SCF, sulbactam + cefoperazone; sSSTIs, superficial skin and soft tissue infections; SXT, trimethoprim–sulfamethoxazole; TZP, tazobactam + piperacillin.

The MDR prevalence of *E. coli, Klebsiella* spp., *P. aeruginosa*, and *S. aureus* was 70%, 75%, 58%, and 73% respectively. Overall, the prevalence of *E. coli* and *Klebsiella* MDR among dSSTIs was higher than that in *P. aeruginosa* and *S. aureus*, which was higher among sSSTIs **(Table 3)**.

**Table 3.** MDR pattern of bacteria isolated from sSSTIs and dSSTIs

**Bacterial isolate**	**SSTIs**	**Antimicrobial classes related to number (%)**					

		***R*_1_**	***R*_2_**	***R*_3_**	***R*_4_**	**≥*R*_5_**	**MDR, n (%)**
*E. coli*	sSSTI (n = 57)	8 (14)	8 (14)	14 (25)	6 (11)	14 (25)	34 (60)
	dSSTI (n = 69)	4 (6)	8 (12)	21 (30)	10 (14)	23 (33)	54 (78)
*Klebsiella spp.*	sSSTI (n = 27)	2 (7)	4 (15)	5 (19)	4 (15)	9 (33)	18 (67)
	dSSTI (n = 49)	2 (4)	4 (8)	9 (18)	5 (10)	25 (51)	39 (80)
*P. aeruginosa*	sSSTI (n = 9)	3 (33)	0 (0)	1 (11)	0 (0)	5 (56)	6 (66.6)
	dSSTI (n= 29)	4 (14)	5 (17)	5 (17)	2 (7)	9 (31)	16 (55)
*S. aureus*	sSSTI (n = 14)	0 (0)	1 (7)	7 (50)	3 (21)	2 (14)	12 (86)
	dSSTI (n = 43)	2 (5)	9 (21)	21 (49)	4 (9)	5 (12)	30 (70)

dSSTIs, deep-seated skin and soft tissue infections; MDR, multidrug resistance; sSSTIs, superficial skin and soft tissue infections.

## Discussion

In this study, mono-bacteria were isolated from 78% of patients, of which 67% were males. Similar results have been reported from Pakistan and Ethiopia with the single pathogen isolation (77% and 82%, respectively) with male dominance [11, 7]. In the present study, the incidence of SSTIs was higher (45%) among patients in the 21–50 years age group; this is in agreement with previous studies that report higher prevalence in the 15–44 years age group [11, 7]. Moreover, the rate of SSTIs was higher among inpatients (61%) in our study, which is analogous to a previous study (72.8%) [7]. In this study, dSSTIs were more common (64%), among which abscess and surgical sites were the major sites (94%), whereas wound was the most common site (66%) for sSSTIs. This was in agreement with a similar study from Pakistan, where abscess was the most common site [11]. Chronic kidney disease and diabetes mellitus were major underlying conditions (64%) in our patients. A previous study has reported diabetes mellitus, chronic kidney disease, and heart failure as the most common underlying medical conditions [8].

In the current study, the GNR/GPC ratio was 4:1. Furthermore, the prevalence rate of GNR was higher (79%) than reported in the studies conducted in India, Ethiopia, and Greece (57%, 57%, and 55%, respectively) [7,8,9]. The most frequent GNRs in this study were *E. coli* (36%), followed by *Klebsiella* spp. (22%) and *P. aeruginosa* (11%). The results are in concordance with a study from Pakistan that reported *E. coli* and *P. aeruginosa* from complicated SSTIs as the most frequent GNRs. Another study from India reported *P. aeruginosa* (21%) and *E. coli* (17.5%) as predominant GNRs [9]. This deviance in the isolation may be due to geographical variation or study design. *S. aureus* was the predominant (16%) GPC found in this study, which is in agreement with studies published from Pakistan and Nepal [11, 20].

In our study, >65% of all 3 GNRs revealed resistance against each oral antibiotic tested. This was in agreement with a similar study from Nepal that reported the majority of *E. coli* isolates were resistant to amoxicillin and co-trimoxazole and all *E. coli* isolates were resistant to ciprofloxacin [20]. A recent review from Pakistan reported the resistance pattern of *E. coli* against ciprofloxacin (58%–74%), co-trimoxazole (65%–82%), and co-amoxiclav (40%–92%), whereas the resistance among *Klebsiella* spp. was reported against ciprofloxacin (24%–86%) and co-trimoxazole (58%–69%) [17].

For injectable antibiotics, only a least number of GNRs were resistant against amikacin and a highest number were resistant against ceftriaxone in our study. We report >79% of *E. coli* and *Klebsiella* spp. were resistant against ceftriaxone. A similar high resistance rate of >80% of *Klebsiella* spp. and *E. coli* against third-generation cephalosporins has been reported [9, 11]. *E. coli* was found the least resistant against amikacin (9%) in this study., which is in contrast to other studies where they have ascertained a very high resistance rate among *E. coli* against amikacin (63%–80%). For *Klebsiella* spp., resistance against amikacin was 38% in our study, which is similar to the resistance reported in a study from India (40%) and higher than that reported in a study from Pakistan (25%) [9, 11]. This indicates that resistance against amikacin among *Klebsiella* spp. is increasing in Pakistan. In this study, 39% of *P. aeruginosa* demonstrated resistance against amikacin, which is lower than that reported in studies from Pakistan (66.6%) and India (47.8%) [9, 11].

In contrast to a previous study from Pakistan where resistance against TZP among *Klebsiella* spp., *P. aeruginosa*, and *E. coli* was reported 12.5%, 6%, and 2%, respectively. Our findings demonstrate a much higher rate of resistance, i.e., 43%, 34%, and 29%, respectively. This significant increase in resistance in our isolates may be due to the overuse of antibiotics in our hospital. However, our results are similar to those of a study from India where high resistance prevalence among *E. coli* (63%) and *Klebsiella* spp. (60%) against TZP has been reported [9, 11]. In our study, the rate of resistance among *P. aeruginosa*, *Klebsiella* spp., and *E. coli* to SCF was 42%, 49%, and 30%, respectively.

In this study, a very high level of resistance against carbapenem was found among *P. aeruginosa* (53%), *Klebsiella* spp. (32%), and *E. coli* (18%) in contrast to a previous studies from Pakistan and India where low resistance among *Klebsiella* spp. (0% and 0%, respectively), *P. aeruginosa* (8.4% and 8.6%, respectively), and *E. coli* (1.7 and 2.6%, respectively) has been reported. Another report form the USA and Europe also reported all *E. coli* isolates were sensitive to imipenem [21]. This high level of resistance against carbapenem in our region may be due to the increasing expression of extended spectrum beta-lactamases, the use of self-medication, and indiscriminate use of antibiotics. In our study, all isolates of *P. aeruginosa* and *S. aureus* were sensitive to polymyxin B and vancomycin, respectively. This may be accredited to the presence of intrinsic genes among these isolates [22].

We noticed differences in the resistance pattern of isolates from dSSTIs and sSSTIs. A comparatively high number of *E. coli* and *Klebsiella* spp. isolates were resistant from dSSTIs against oral, injectable, and carbapenem antibiotics, whereas *S. aureus* and *P. aeruginosa* isolated from sSSTIs were more resistant.

The cumulative resistance rate of bacteria from hospitalized patients was higher in our study, indicating the circulation of highly resistant bacteria, particularly MDR *E. coli, Klebsiella* spp., and *S. aureus* within the hospital environment, which alarms for continuous monitoring and isolation of patients from immunocompromised and health care workers. This was in agreement with the previous reports where a higher proportion of antimicrobial resistance among inpatients has been reported [23].

The predominant GPC in our study was *S. aureus* (78%). Notably, a high prevalence of MRSA (86%) was ascertained in our study population compared to that in previous studies from Pakistan (53%, 36.1%, and 42%) [24,25,26]. A meta-analysis of 37 studies from China has reported the pooled prevalence of MRSA (21.2%) [27]. This strikingly high frequency of MRSA might be attributed to the frequent use of empirical treatment for SSTIs, a paradigm shift in the daily life style due to the COVID-19 pandemic, or a typical public hospital with overuse of intravenous drugs. In our study, 74% of *S. aureus* were multidrug-resistant, which is lower than that reported in a previous study from Ethiopia 94.8% [20].

The high prevalence of SSTIs was found among males in the 21–50 years age group. The predominant isolates were *E. coli, Klebsiella* spp., *P. aeruginosa*, and *S. aureus*. More than 50% of all GNRs were resistant to each oral antibiotic. For intravenous antibiotics, the least resistance was determined against amikacin, and the highest was determined against ceftriaxone. For carbapenem, *P. aeruginosa* showed the highest resistance (53%). *S. aureus* showed the highest resistance against ciprofloxacin, and the least resistance against clindamycin. MDR *E. coli* and *Klebsiella* spp. isolates were found to be predominant among dSSTIs. We recommend continuous surveillance for appropriate antibiotic usage and for preventing the emergence of MDR pathogens.
